# Value for health: how fortified infant cereals provide cost-effective solutions to iron deficiency anaemia in Egypt

**DOI:** 10.3389/fnut.2025.1570683

**Published:** 2025-07-15

**Authors:** Seham Elmrayed, Sara Colombo Mottaz, Livia Dainelli, Helmy Salib, Hossam Abdel Ghaffar, Yasmin Gamal El Gendy

**Affiliations:** ^1^Institute of Global Health and Human Ecology, American University in Cairo, Cairo, Egypt; ^2^Nestlé Nutrition, Department of Medical, Scientific and Regulatory Unit, Vevey, Switzerland; ^3^Nestlé Health Science, Department of Global Market Access & Pricing Lead, Lausanne, Switzerland; ^4^Nestlé Nutrition, Department of Medical, Scientific Affairs, Cairo, Egypt; ^5^Government of Egypt, Egyptian Ministry of Health and Population, Cairo, Egypt; ^6^Department of Pediatrics and Clinical Nutrition, Ain Shams University, Cairo, Egypt

**Keywords:** iron deficiency anaemia, dietary supplements, fortified infant cereals, health economics, cost-effectiveness analysis

## Abstract

**Background:**

Anaemia prevalence among Egyptian children under 5 years of age increased from 27.2% in 2014 to 43% in 2024, primarily attributed to iron deficiency anaemia (IDA). The World Health Organization and the United Nations International Children's Emergency Fund recommend iron-fortified foods and supplements to combat IDA. In the absence of longitudinal data among Egyptian children with anaemia, a microsimulation and cost-effectiveness analysis was conducted to evaluate the economic and health impacts of consuming iron-fortified cereals (IFC) in reducing IDA prevalence among Egyptian children under 2 years of age.

**Methods:**

Data of 1707 children under 2 years of age from Egyptian Family Health Survey 2021 (EFHS) were used to create a virtual cohort of 100,000 through Monte Carlo simulations, stratified by age, gender, wealth index, and anaemia severity. A Markov model projected transitions in anaemia severity over 10 years for IFC and non-IFC consumers. Costs for IFC and home-based foods were derived from market research and existing literature, with cost-effectiveness evaluated using the incremental cost-effectiveness ratio (ICER), indicating the additional cost required to gain one additional unit of effectiveness (in our case the disability-adjusted life years [DALYs]) when two approaches are compared.

**Results:**

The per-day cost of home-based food was 0.37 United States Dollar (USD) per child, with an additional 0.17 USD for IFC consumers. Based on 5% IFC consumption (EFHS 2021) anaemia prevalence was projected to reduce to 32% over 10 years. DALYs averted among IFC consumers were 0.006 DALY/day and 22 DALYs over a period of 10 years. The obtained ICER of −4.14 suggests that an IFC intervention can be more effective and less costly than no intervention.

**Conclusion:**

IFC interventions among Egyptian children under 2 years of age are crucial for reducing IDA. IFC consumption lowers DALYs and offers significant cost savings over 10 years, making it an effective health and economic strategy. With 4.058 million children under 2 years of age in Egypt, IFC interventions could save 7.79 million USD for 1 day of disability averted. This study provides evidence-based policy insight, urging prioritisation of IFC recommendation in public health strategies to combat IDA in children and reduce economic burdens.

## 1 Introduction

Micronutrient deficiencies during early childhood (before 2 years of age) can significantly hinder a child's physical growth and neurodevelopment, leading to short- and long-term health issues with social and economic repercussions later in life (1, 2). This is particularly concerning for iron deficiency anaemia (IDA), which affects 40%−50% of the global population and 41.4% of children under 5 years of age (3, 4). Anaemia can impair cognitive and physical development, increase infant mortality and morbidity, and reduce adult work productivity, resulting in sociocultural and economic impacts (2). The prevalence of anaemia among children in the Eastern Mediterranean region ranges between 23% and 60% in various population subgroups (5). In Egypt, the prevalence of anaemia among children increased from 27.2% in 2014 to 43% in 2024 (6, 7), regardless of the free paediatric iron supplementation programme supported by the local government. The 2021 Egyptian Family Health Survey (EFHS) indicated that 20.9% of children under 5 years of age were affected by mild anaemia, 20.8% by moderate anaemia, and 1.4% by severe anaemia (8).

To ensure appropriate dietary intake and prevent deficiencies in children, the World Health Organization (WHO) and the United Nations International Children's Emergency Fund recommend initiating complementary feeding after 6 months of age, incorporating micronutrient-fortified foods to reduce the risk of micronutrient deficiencies, including IDA (9). To prevent IDA, the WHO advises dietary fortification of 2 mg/kg body weight and daily iron supplementation for children under 5 years of age (9, 10). However, adherence to daily iron supplements is frequently suboptimal among children due to factors such as taste, palatability, forgetfulness, and cultural considerations (9, 11). Universal access to iron-rich foods in the child's early years is a crucial public health intervention to support children's growth and development, building a more productive society (4). This study was conducted to evaluate the economic and health impact of consuming iron-fortified cereals (IFC) in reducing IDA prevalence among Egyptian children under 2 years of age.

## 2 Methods

### 2.1 Data source and study population

The base data for this study were sourced from the recent EFHS 2021 survey, a nationally representative survey, in which data from 1707 children aged 6 months to 2 years were analysed to assess haemoglobin (Hb) levels and IFC consumption (8). The EFHS 2021 survey was selected as it provides comprehensive information on dietary practises, including consumption of IFC, across various regions of Egypt. Although the virtual cohort used in the modelling study is based on a subset of 1,707 children from this survey (as base data), the broader EFHS 2021 sample was designed to reflect Egypt's national-level demographic diversity, including both urban and rural populations. Key variables included age, gender, home-based infant feeding practises (types of food, breastfeeding frequency, formula feeding, and complementary foods), access to IFC (availability and ability of caregivers of infants and young children to obtain and utilise IFC), maternal dietary practises, and relevant social and cultural factors. A multivariate analysis was conducted to adjust for any confounding factors. To determine the significant difference in Hb levels among all the key variables, a *post hoc* analysis with Bonferroni correction was conducted.

Anaemia was defined as an Hb concentration below 11 g/dL and was further categorised into mild anaemia (Hb 10–10.9 g/dL), moderate anaemia (Hb 7–9.9 g/dL), and severe anaemia (Hb < 7 g/dL) (8). Additionally, children were stratified into socioeconomic groups based on a wealth index, categorising households as poorest, poor, middle, upper-middle, and wealthiest. Home-based foods were grouped into a single category hence dietary variations were not considered in the microsimulation model.

### 2.2 Microsimulation study

**Figure 1:**
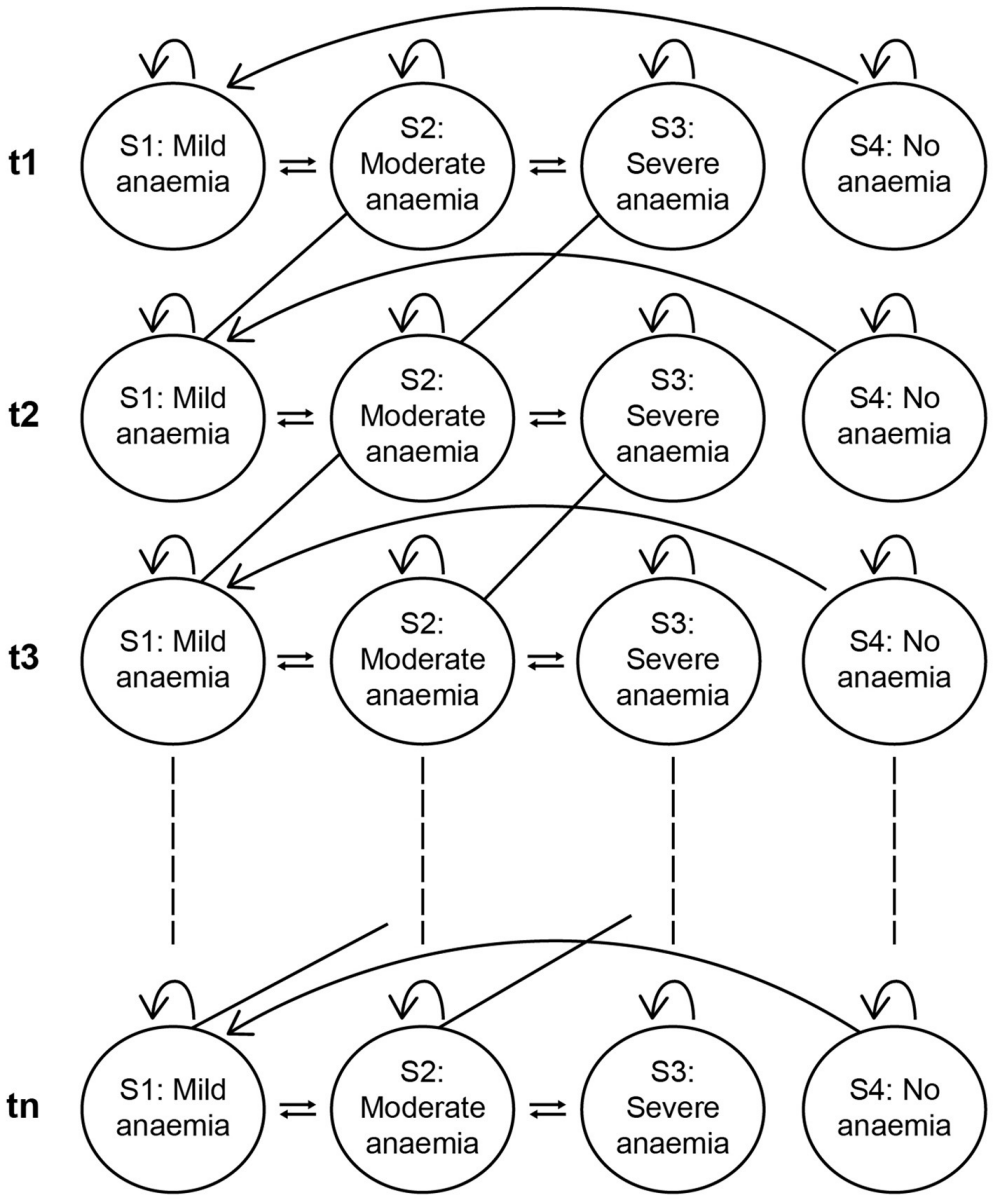
Conceptual model using transition probabilities Markov model.

In the absence of longitudinal population data among Egyptian children with anaemia, a microsimulation analysis followed by a cost-effectiveness analysis (CEA) was conducted to evaluate the impact of consuming IFC during early childhood (6 months to 2 years of age) in reducing the prevalence of IDA and to assess its long-term benefits (estimated for a duration of 10 years). Monte Carlo simulations were used to create a virtual cohort of 100,000 children derived from the initial 1,707 children in the base data. From this, two subgroups were created: Group 1 comprised of children who consumed iron-fortified age-adapted foods as per the base data at least once a day until 2 years of age, with an average iron content of 5.5 g per serving (IFC group) and Group 2 consisted of children not consuming IFC (non-IFC group) (12). Transition probabilities were derived from a combination of empirical data and extrapolation. The extrapolation of data was performed using existing literature. A review of relevant literature was conducted to identify the odds ratio and relative risk of developing anaemia among children in Egypt (2, 13–18). Empirical derivation was based on the rates at which individuals transitioned between health states (19). Equations for transition probabilities for stages of anaemia are presented in Supplementary Table S1 (Equations 1–4). To best account for the transitions between various anaemia states, probabilities for state transitions up to 10 years (6-month intervals) were developed using the Markov model (Figure 1) (19). Each transition probability represented the likelihood of shifting between no anaemia and mild, moderate, or severe anaemia and vice versa at 6-month intervals. The IFC group, consisting of children who received IFC during early childhood (up to 2 years of age), accounting for ~5.6% of boys and 4.3% of girls in the base data for that group range, was compared with children not consuming IFC. Home-based foods, as mentioned in the EFHS 2021 data, were consumed by the children in both groups.

### 2.3 Estimating disability-adjusted life years (DALYs)

DALYs were calculated for 10 years using years of life lost and years lived with disability, incorporating discount rates of 3% (applied after 1 year) to account for the time preference of health benefits (since being healthy now is valued more than being healthy in the future, a discount rate is applied to reduce the value of future health outcomes and reflect the preference for current health) (20). Disability weights for anaemia among children were taken from the Global Burden of Disease 2019 (4).

### 2.4 Estimating incremental cost-effectiveness ratio

The EFHS 2021 survey provided detailed data on feeding practises for children under 2 years of age. To evaluate the cost-effectiveness of IFC in reducing IDA among Egyptian children, the ICER value was calculated, which is a way to compare the costs and benefits of different approaches—IFC consumers vs. non-consumers. The ICER is the incremental change in costs to have IFC divided by the incremental change in health outcome [cost per DALY saved {cost (intervention)-cost (comparator)/DALY averted (intervention)-DALY averted (comparator)}] (21).

The analysis involved comparing the costs and health benefits (Hb levels and anaemia reduction only) over 10 years between children consuming IFC and those not consuming IFC in the first 2 years of their life. Supplementary Table S2 presents the monthly consumption proportions of various home-based foods as reported in the EFHS 2021 (8, 22, 23). The costs of these foods were gathered from multiple sources (22, 23). The costs of semi-solid and solid foods, dairy products (such as yoghurt and cheese), fruits, and vegetables, as well as meat, fish, and poultry products were sourced from the International Food Policy Research Institute (22). Supplementary Table S3 represents the disability weights for anaemia among children aged 6–24 months as per the Global Burden of Disease 2019 (4). Market research data provided the cost estimates for fortified baby foods and infant formulas (23). The duration for IFC consumption by the children was capped at 2 years. Thus, considering 0–6 months as exclusively breastfeeding, IFC consumption starting between 6 and 12 months (considering one timepoint corresponded to a duration of 6 months) will have three timepoints (first consumption timepoint between 6 and 12 months, second consumption timepoint between 12 and 18 months, and third consumption timepoint between 18 and 24 months). Similarly, those between 12 and 18 months will consume IFC at two timepoints (first consumption timepoint between 12 and 18 months, second consumption timepoint between 18 and 24 months) and children between 18 and 24 months will consume IFC at a single timepoint (consumption timepoint between 18 and 24 months).

### 2.5 Sensitivity analysis

To evaluate how uncertainties in the Markov microsimulation model influence ICER per capita per day, both one-way and probabilistic sensitivity analyses were conducted (24). In the one-way sensitivity analysis, every parameter was adjusted individually within a specified range to determine the impact of each parameter on the model outputs in isolation (25). Upper and lower bounds were derived from the minimum and maximum values available in databases. This approach allowed us to observe the impact of each parameter on the model outcomes, enabling the identification of key drivers of cost-effectiveness (26). Additionally, the results of the system model were enunciated through sensitivity analysis, which highlights the significant impacts of uncertainties on system behaviour and performance. For probabilistic sensitivity analysis, model inputs were sampled multiple times from predefined probabilistic distributions. We applied gamma distributions for cost parameters and beta distributions for all other variables. The 95% confidence interval of a specific parameter represents the plausible range of values used in the sensitivity analysis. The model was executed 1,000 times, simulating a cohort of 100,000 children. We then determined how each strategy changed the ICER per capita under various scenarios, stratified for gender and wealth index groups. Median ICER values were reported along with the interquartile range to capture variability.

## 3 Results

The study included 1,707 infants (872 [51.08%] boys and 835 [48.9%] girls) aged 6 months to < 2 years from the EFHS 2021. There was no statistically significant difference in age group distribution between the boys and girls (*p* = 0.430). Among these children, anaemia (Hb levels < 11 g/dL) was observed in 58.6% of children (*n* = 1002), with 24% (*n* = 410) having mild anaemia, 33.6% (*n* = 574) having moderate anaemia, and 1.05% (*n* = 18) having severe anaemia. The difference in anaemia levels between boys and girls was not statistically significant (*p* = 0.604). The mean Hb level was 10.6 ± 1.6 g/dL with no statistically significant difference among boys (10.51 ± 1.63) and girls (10.61 ± 1.57) (*p* = 0.134). Similarly, there was no statistically significant difference among boys and girls when categorised according to the wealth index quintile (*p* = 0.504; Table 1). The results of meta-regression analysis showed non-significant difference among geographic variations (rural/urban); hence, this was not considered as an input parameter for the microsimulation model. The results of *post-hoc* analysis are presented in Supplementary Table S4.

**Table 1:** Description of base data (8).

**Child's age in months**	**All children (1,707) [*n* (%)]**	**Boys (872) [*n* (%)]**	**Girls (835) [*n* (%)]**	***p*-value**
6 to <12	27.48 (469)	28.78 (251)	26.11 (218)	0.430
12 to <18	34.86 (595)	33.83 (295)	35.93 (300)	
18 to <24	31.75 (643)	31.9 (326)	31.62 (317)	
**Anaemia levels**				
0 = Not anaemic	41.30 (705)	41.06 (358)	41.56 (347)	0.604
1 = Mild	24.02 (410)	22.94 (200)	25.15 (210)	
2 = Moderate	33.63 (574)	34.86 (304)	32.34 (270)	
3 = Severe	1.05 (18)	1.15 (10)	0.96 (8)	
Haemoglobin level (g/dL) (mean ± SD)	10.56 ± 1.60	10.51 ± 1.63	10.61 ± 1.57	0.134
**Wealth index quintile**				
1 = Lowest quintile (poorest)	17.51 (299)	17.78 (155)	17.25 (144)	0.504
2 = Second quintile (poor)	18.92 (323)	20.18 (176)	17.60 (147)	
3 = Third quintile (middle)	21.56 (368)	21.22 (185)	21.92 (183)	
4 = Fourth quintile (upper middle)	23.61 (403)	23.74 (207)	23.47 (196)	
5 = Highest quintile (wealthiest)	18.39 (314)	17.09 (149)	19.76 (165)	
**Feeding practises**				
Children consuming fortified infant cereals (baby foods)	85 (5.0%)	49 (5.6%)	36 (4.3%)	0.211
Receiving subsidies for infant formula (*n =* 248)	100 (5.86%)	51 (5.85%)	49 (5.87%)	0.960
Availability of infant formula at local PHC clinic (*n =* 226)	103 (6.03%)	55 (6.3%)	48 (5.75%)	0.569
Children eating grains and cereal-based foods	1,258 (73.7%)	633 (72.6%)	625 (74.9%)	0.308

PHC, Primary healthcare centre; SD, Standard deviation.

### 3.1 Cost of the intervention

Based on the dietary practises highlighted under the EFHS, the average cost of home-based foods per child per day (below 2 years) was estimated as 0.37 United States Dollar (USD) and the average cost of IFC per child per day was identified as 0.17 USD. Thus, the cost of consuming IFC along with home-based foods for each child was around 0.54 USD (Table 2). This means that the total cost of the intervention is equal to a maximum of USD 291 for children who will start the consumption of IFC at 6 months and will finish at 2 years old and equal to USD 199.8 for those who will not consume IFC.

**Table 2:** Cost-effectiveness analysis for IFC among IFC consumers vs. non-consumers.

**Indicators for cost effectiveness**	**Boys**	**Girls**
DALY with IFC per day (in 10 years)	0.021 (77)	0.021 (77)
DALY without IFC per day (in 10 years)	0.027 (99)	0.027 (99)
Cost of food per person/day with IFC (for 18 months)	0.540 (291) USD	0.540 (291) USD
Cost of food per person/day without IFC (for 18 months)	0.370 (199.8) USD	0.370 (199.8) USD
ICER 10 years	−4.14.	−4.14

DALY, Disability-adjusted life years; ICER, Incremental cost-effectiveness ratio; IFC, Iron-fortified cereals; USD, United States Dollar.

### 3.2 Effects of iron fortification on anaemia

**Figure 2:**
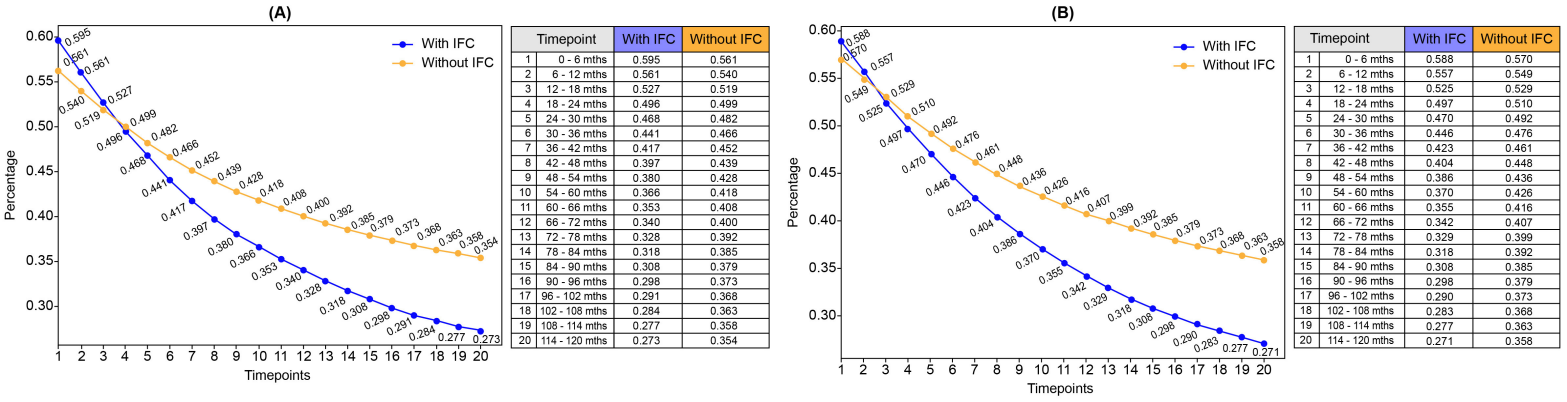
Trends in population prevalence of anaemia among consumers and non-consumers of IFC over 10 years. **(A)** Prevalence of anaemia over 10 years: Girls. **(B)** Prevalence of anaemia over 10 years: Boys. IFC, Iron-fortified cereals.

Based on the transition between mild, moderate, and severe anaemia, changes are anticipated in the prevalence of anaemia in the next 10 years (Supplementary Tables S5, S6; Figure 2). At baseline (timepoint t1), extrapolated from the EFHS data, the anaemia prevalence in the population consuming IFC along with home-based foods was almost similar in both groups (with no gender disparities): around 59% among IFC consumers vs. 57% among non-IFC consumers. It is important to note that the EFHS lacks information on the consumption pattern of IFC. By the end of the simulated period (timepoint t20), anaemia prevalence decreased to 27% in the IFC group, while it remained higher at 36% in the non-IFC group. Across all time points, the IFC group consistently exhibited a lower anaemia prevalence than the non-IFC group (Supplementary Tables S5, S6).

### 3.3 Effects of anaemia on DALYs

Both boys and girls who consumed IFC along with home-based foods in their initial years of life (<2 years of life) experienced a lower number of days lost due to disability (for example, fatigue, weakness, and shortness of breath, making activities of daily living difficult) arising from IDA compared to the girls and boys who did not consume IFC (4). The days lost to disability due to IDA in a child consuming IFC were 77 days (0.021 DALY), while the days lost in a child not consuming IFC were 99 days (0.027 DALY). DALYs averted among IFC consumers compared with non-IFC consumers were 0.006 DALY/day. Thus, IFC consumers will save around 22 days of disability arising from IDA in 10 years, indicating that IFC contribute to improved health outcomes by preventing or reducing the severity of anaemia.

### 3.4 Cost-effectiveness analysis of iron fortification

This study highlighted that an 18-month intervention with IFC leads to an ICER equal to −4.14 USD over a 10-year period (Table 2). When using DALYs, a negative ICER can occur if either the numerator (incremental cost) or the denominator (DALYs averted) is negative. Negative cost (numerator) implies that the new intervention is less costly than the reference intervention. However, this is not the case in the present model: fortified cereals come with an additional cost. In the present model, we have incremental costs (positive numerator) and negative DALYs (denominator). This means that the new intervention leads to fewer DALYs (more health gains) than the reference intervention (no consumption of fortified cereals). Among children consuming IFC, the total days saved from disability were 2.19 days per annum. The cost of saving 1 day of disability was 1.92 USD.

### 3.5 Evaluating model robustness

One-way sensitivity analysis demonstrated that variations in the cost of IFC had the most significant impact on ICER (Supplementary Figures S1A, B). A 10% reduction in the cost of IFC shifted the median ICER from a cost-saving value of −29.8 USD per capita per day to a positive value of 5.09 USD, with a wide interquartile range, indicating substantial uncertainty. In contrast, a 20% reduction in cost yielded a median ICER of 5.63 USD with a narrower range, suggesting more stable but still mixed outcomes. Subgroup analyses revealed that children from the poorest wealth index had the most favourable ICERs, indicating consistent cost savings. Conversely, the wealthiest subgroup displayed a wider range, with potential for both cost savings and modest cost increases. Gender differences had a minimal influence on the ICER, closely aligning with the base case. These findings strongly underscore that cost parameters and socioeconomic strata are crucial determinants of the model's outcomes and should be key considerations in policy implementation. The cost-effectiveness acceptability curve shows an 80% probability that IFC are a cost-effective intervention at an additional cost of 0.17/day USD, increasing with cost reductions (10%−20% cost reduction) but dropping to 40% with no funding support. This demonstrates that affordability and willingness-to-pay (WTP) thresholds critically determine the intervention's cost-effectiveness (Supplementary Figure S1B).

## 4 Discussion

The study findings suggested a significant reduction in the prevalence of anaemia from 59% to 27% over 10 years among both boys and girls who consumed IFC at least once a day until 2 years of age. IFC intervention was considered for children under 2 years of age because the first 1,000 days (from conception to 24 months) is the most critical window for physical and cognitive development. The majority of the linear growth deficits that make up the under-5 stunting burden are accumulated during this time, reflecting nutritional deficiencies (27, 28). These findings emphasise the critical role of iron food fortification, specifically IFC, in early childhood in mitigating IDA and the related long-term consequences.

The current study is consistent with previous research. Harrison et al. (18) reported a significant reduction in the prevalence of IDA among infants in Ghana who consumed micronutrient-fortified cereals, providing 3.75 mg of iron as ferrous fumarate per 50 g of cereal. Daily consumption varied by age: 50 g/day for infants aged 6–8 months, 75 g/day for those aged 9–11 months, and 100 g/day for those aged 12–18 months. In the fortified cereal group, the prevalence of IDA decreased from 81.1% to 42.8%, compared to a reduction from 89.1% to 62.8% in the non-fortified group (*p* = 0.006) (18). Similarly, Moumin et al. (29) conducted a study in Australia involving infants aged 6–12 months who were given 18 g (300 kJ) of iron-fortified infant cereal per serving. This intervention significantly reduced the prevalence of iron inadequacy from 75% to 5%. Furthermore, Ekoe et al. (17) reported a significantly lower prevalence of IDA (14.6%) among children aged 18–59 months receiving micronutrient-fortified cereals compared to 53.4% in those who did not receive the fortified cereals (*p* < 0.001). Collectively, these studies reinforce the positive impact of iron fortification in reducing anaemia prevalence and improving iron status in young children, highlighting the efficacy of such interventions across different populations and settings.

Many studies show that iron supplementation results in a 58%−72% reduction in anaemia prevalence, compared to a 23%−62.8% reduction in children consuming IFC (14, 15, 18). Although the reduction in anaemia prevalence is higher among children consuming iron supplementation, iron supplementation often encounters issues such as poor compliance (9). Moreover, supplementing iron in iron-replete infants or those with unknown iron status can negatively impact growth and weight gain (9). IFC have better compliance and offer a preventive, long-term, sustainable approach. This strategy, when combined with home-based foods, can effectively meet nutritional needs during the critical period of 6–23 months of age (17). IFC were also associated with enhancements in neurodevelopmental outcomes. Specifically, children consuming IFC showed significantly higher scores on the Bayley Scales of Infant and Toddler Development, Third Edition, in areas such as language (*p* = 0.003), motor development (*p* = 0.018), and social-emotional (*p* = 0.004) and adaptive behaviour (*p* < 0.001), though not in cognitive development (*p* = 0.980), compared to those not consuming IFC (15). While individual studies have reported these positive health outcomes, extrapolation of these data over a 10-year period is not feasible due to the overlapping nature of micronutrient deficiencies and health outcomes. Aggregating the burden of micronutrient deficiencies from individual studies may lead to overestimation (Supplementary Table S7) (2). For all these reasons, these additional health outcomes were excluded from the current analysis.

Although this study considered IFC intervention up to 2 years of age, long-lasting effects in improving health status are expected. These findings indicate that regular consumption of IFC containing 5.5 g of iron per serving at least once daily during the first 2 years of life along with home-based foods results in a substantial reduction in anaemia prevalence over time and is an efficacious public health strategy in reducing anaemia prevalence. While IFC incur a small increase per-day household food cost, adding 0.17 USD to the baseline 0.37 USD for home-made foods, this reflects only the direct cost of food and does not capture the broader economic implications of anaemia-related health outcomes. In the current CEA, where the cost of the intervention is higher than the comparator but the DALYs of the intervention are lower than the comparator, it means that the intervention is more expensive, but it also results in better health outcomes (fewer DALYs). In other words, the DALYs gained (health benefits) come at an additional cost. Thus, in such cases, the negative ICER (−4.14 USD) cannot be directly interpreted. Hence to calculate the cost-effectiveness of the current intervention, WTP threshold is considered. This means comparing the cost per DALY averted (4.14 USD in the current study) independently from its sign (negative in this case) to the country's gross domestic product (GDP) per capita or policy norms. If the intervention's additional cost is < 1 GDP per capita, it can be considered cost-effective. If the cost per DALY averted is below the WTP threshold, the intervention is cost-effective despite its higher cost. If the cost per DALY averted exceeds the WTP threshold, the intervention is not cost-effective (30). In this study, given the Egyptian daily GDP per capita equal to 6.6 USD (31, 32), the intervention can be considered cost-effective. As per WHO-CHOosing Interventions that are Cost-Effective (CHOICE), the current IFC intervention can be considered as highly cost-effective as the ICER is <1 × GDP per capita per DALY averted (33, 34).

For the total population of Egyptian children under 2 years of age, which is 4.058 million (35), the IFC intervention is expected to be cost saving, with an estimated savings of ~7.79 million USD (1.92 ^*^ 4,058,000) for each day of disability averted. This suggests that the intervention is not only effective and less costly over a 10-year period but also holds significant policy implications. By demonstrating economic benefit, it highlights the importance of incorporating such interventions into comprehensive public health strategies to enhance outcomes for young children while ensuring efficient resource utilisation. A common method to assess cost-effectiveness is to compare the ICER to a WTP threshold. Since the ICER for the IFC intervention is negative, it is lower than the standard cost-effectiveness threshold, including Egypt's per capita daily income of 6.6 USD (31, 32). This suggests that IFC is a cost-effective intervention, providing health benefits at a lower cost than what is typically considered affordable or sustainable based on the country's average income. Although the intervention involves a higher upfront cost for food, it leads to net savings by alleviating the health burden and reducing the broader economic impact of anaemia. The analysis accounts for total costs, encompassing healthcare expenses, productivity losses, and the long-term economic consequences of anaemia. By effectively reducing anaemia prevalence, IFC is associated with fewer healthcare visits, reduced treatment costs, and improved productivity. These savings have the potential to offset or exceed the additional daily cost. Furthermore, the analysis spans over 10 years, allowing the cumulative benefits of reduced anaemia prevalence to outweigh the modest increase in daily food costs.

However, several barriers to the consumption of IFC persist. These include inadequate caregiver awareness and nutritional education on the benefits and proper use of IFC along with cultural beliefs favouring traditional unfortified dietary practises. Furthermore, accessibility issues in rural or underserved areas, gaps in healthcare provider support, potential inadequacies in government policies to implement national-level strategies for micronutrient intervention such as IFC consumption, lack of subsidy programs, weak monitoring and evaluation systems, under-resourced health information infrastructure, and supply chain inefficiencies contribute to these challenges (27). Addressing these barriers is essential for optimising IFC utilisation and improving child health outcomes. To scale up the distribution of IFC, a collaborative approach involving strengthening the health system and multisectoral collaboration among government, public health agencies, and healthcare providers can be implemented. The distribution of IFC can be integrated into existing maternal and child health programs thereby utilising their infrastructure to reach a larger population (28). Public–private partnerships with food manufacturers will help increase the production, affordability, and availability of fortified cereals. Additionally, awareness campaigns can be implemented, with training for health workers and community leaders to educate families on the benefits of iron fortification. The Global Alliance for Improved Nutrition has also recognised the advantages of fortified micronutrient foods in addressing deficiencies. However, it has been reported that, on average, only 50% of fortified foods meet national standards in low- and middle-income countries like Egypt (36). To maximise their effectiveness and be available to a wider population, IFC awareness and accessibility programmes need to be enhanced.

Implementing IFC intervention in children is crucial from a societal perspective. IFC benefits both families and the healthcare system by improving child health outcomes and reducing hospital-related costs. This will lead to lower healthcare resource utilisation and economic savings for families and society as a whole. Additionally, it is also essential from a policy perspective, as the study provides evidence for policymakers to prioritise IFC interventions and guide cost-effective public health strategies.

This microsimulation study has some limitations. For instance, the analysis did not fully account for the influence of confounding factors, which may affect the association between early childhood iron deficiency and long-term health and economic outcomes. Additionally, the model's estimates may be affected by the presence of other micronutrient deficiencies and multiple diseases that can contribute to IDA, introducing additional complexity and uncertainty to the findings of this model. The assumptions in the Markov modelling may not accurately reflect real-world variations. An unobserved heterogeneity can potentially impact the outcomes of the Markov modelling. Additionally, the absence of direct, longitudinal observational data from Egyptian children and relying solely on data collected from the EFHS 2021 restricts the generalisability of the findings. Furthermore, cost estimates based on market research may introduce bias, potentially impacting the accuracy of the economic outcomes. Despite these limitations, this health economic model strategy serves as a valuable tool for assisting public health professionals in evaluating both the effectiveness and cost-efficiency of various interventions.

## 5 Conclusion

This study highlights that regular consumption of IFC during early childhood is a cost-effective solution to mitigate IDA which is associated with long-term personal and societal negative implications. Despite the benefits demonstrated, barriers such as insufficient awareness, cultural preferences, and accessibility issues remain. Addressing these challenges is imperative for reducing the prevalence of iron deficiency and its extensive impact on child health while mitigating the associated health and economic burdens. Government bodies should enhance the availability and accessibility of fortified age-adapted foods and ensure compliance with quality standards. Paediatric associations should integrate iron-fortified foods into clinical and complementary feeding guidelines, and healthcare providers must educate caregivers on the short- and long-term benefits of fortified foods in early childhood.

## Data Availability

The original contributions presented in the study are included in the article/Supplementary material, further inquiries can be directed to the corresponding author.
