# Supplementary material for: Value for health: how fortified infant cereals provide cost-effective solutions to iron deficiency anaemia in Egypt
Source: Front Nutr. 2025 Jul 15;12:1570683. doi: 10.3389/fnut.2025.1570683 (PMC12305812; doi:10.3389/fnut.2025.1570683)
Supplement: Supplementary file 1 [file Data_Sheet_1.docx]

Supplementary Material

**Supplementary Table S1:** Equations for transition probabilities for stages of anaemia

| **Sr. No.** | **Equations** |
| --- | --- |
| Equation 1 | $RR=\frac{pr1}{pr0}$ |
| Equation 2 | $\text{p}\text{1}=RR X pr0 i.e, =\frac{pr1}{pr0}X pr0$  *pr1: Probability in exposed (anaemia, iron-fortified cereals, etc.); pr0: Probability in unexposed (normal, no IF cereals, etc.); RR: Relative risk* |
| Equation 3 | pr_tot_$=\left( p0 X pr0 \right)+(p1 X pr1)+(p2 X pr$2)  *p: Known proportion of anaemic children at t0, t1, t2; prtot: Known probability of anaemia at given time points* |
| Equation 4 | pr_tot_$=pr0 X \left( p0+(p1 X\frac{pr1}{pr0} \right)+\left( p2 X\frac{pr2}{pr1} \right)\ldots$  or,  pr_tot_$=pr0 X \left( p0+(p1 X RR1 \right)+\left( p2 X RR2 \right)\ldots$  *RR1: Relative risk in exposed (anaemia, iron fortified cereals, etc.); RR2: Relative risk in unexposed (normal, no IF cereals, etc.)* |

# Supplementary Tables

**Supplementary Table S2:** Child feeding practices among children less than 2 years of age in Egypt (8,22–4)

|  | **Liquids** | | | **Solid or semisolid foods** | | | | | | | | | |  |  |  |
| --- | --- | --- | --- | --- | --- | --- | --- | --- | --- | --- | --- | --- | --- | --- | --- | --- |
| **Age in months** | **Infant formula** | **Other milk** | **Other liquids** | **Fortified baby foods** | **Food made from grains** | **Fruits and vegetables rich in vitamin A** | **Other fruits and vegetables** | **Food made from roots and tubers** | **Food made from legumes and nuts** | **Meat, fish, poultry** | **Eggs** | **Cheese, yoghurt, other milk products** | **Any solid or semisolid food** | | **Number of children** |  |
| **Cost of food per day (USD)** | **26.31 *#** | **0.57 *#** |  | **5.1**  **##** | **1.12*** | **1.23*** | **0.77*** | **1 *** | **2.31 *** | **0.3 **** | **1.21 *#** | **12.6 **** | **0.17 **** | |  |  |
| **Percentage of breast-feeding children (Egyptian Family Health Survey)** | | | | | | | | | | | | | | | | |
| 0–1 | 18.1 | 4.1 | 10 | 0.7 | 4.9 | 5.1 | 3.6 | 4.6 | 3.3 | 6 | 2 | 3.3 | 10.5 | | 267 |  |
| 2–3 | 21.4 | 9.1 | 10.8 | 1.5 | 2.8 | 0.5 | 1.7 | 1.6 | 0.9 | 2.2 | 0.3 | 2.2 | 7.7 | | 349 |  |
| 4–5 | 18.5 | 8.5 | 13.8 | 4.8 | 13.8 | 5.3 | 4.6 | 7.8 | 4 | 5.4 | 4.6 | 16.8 | 32.9 | | 297 |  |
| 6–8 | 12.1 | 12.2 | 35.4 | 12.2 | 38.9 | 17.7 | 25.4 | 26.6 | 13.2 | 15.8 | 12.7 | 38 | 70.2 | | 408 |  |
| 9–11 | 8 | 12 | 50.4 | 5.6 | 67.7 | 29.8 | 40.4 | 39.3 | 26.5 | 36.5 | 24.3 | 52.6 | 87.7 | | 443 |  |
| 12–17 | 4.5 | 14.9 | 60.2 | 4.5 | 77.4 | 36.5 | 54.6 | 51 | 37.8 | 53.7 | 31.1 | 58.9 | 94.6 | | 822 |  |
| 18–23 | 3.1 | 19.9 | 65.2 | 2.5 | 89.4 | 45.5 | 63.4 | 56.5 | 39.6 | 59.8 | 32.8 | 57.8 | 96.4 | | 295 |  |
| 6–23 | 6.7 | 14.4 | 53.6 | 6 | 69 | 32.5 | 46.7 | 44.1 | 30.4 | 42.9 | 26 | 53 | 88.2 | | 1968 |  |
| **Percentage of nonbreast-feeding children (Egyptian Family Health Survey)** | | | | | | | | | | | | | | | | |
| 2–3 | 41.4 | 33.2 | 27.6 | 2.6 | 2.6 | 0.2 | 0.2 | 0 | 0 | 0 | 0 | 6.9 | 7.1 | | 46 |  |
| 4–5 | 46 | 25.6 | 18.7 | 10.6 | 15.8 | 4.1 | 7.8 | 5 | 1.1 | 8.6 | 7 | 16.1 | 35 | | 57 |  |
| 6–8 | 68.7 | 32.1 | 43.5 | 16.9 | 43.9 | 16.4 | 20.1 | 17.2 | 15.4 | 18.1 | 10.7 | 40.8 | 64.3 | | 79 |  |
| 9–11 | 45.1 | 38.6 | 59 | 12 | 73.7 | 37.2 | 45.6 | 48.1 | 26.4 | 42 | 28.5 | 55.7 | 92.1 | | 55 |  |
| 12–17 | 21.2 | 42.6 | 70 | 6.7 | 82.9 | 43.3 | 57.3 | 50.8 | 33.4 | 60.1 | 30.4 | 62.1 | 96.3 | | 365 |  |
| 18–23 | 3.8 | 27.4 | 71.4 | 2.3 | 88.7 | 52.4 | 64.8 | 55 | 41.9 | 62.8 | 36.1 | 62.4 | 98.9 | | 810 |  |
| 6–23 | 14.3 | 32.4 | 68.8 | 4.8 | 83.7 | 47 | 59.2 | 51.3 | 37.3 | 58.5 | 32.7 | 60.7 | 95.8 | | 1308 |  |

*#CEIC. China Retail Price: 36 City Avg: Milk Powder: Infant Formula: Stage 3: 400 g: Pack: Import (2024). https://www.ceicdata.com/en/china/price-monitoring-center-ndrc-36-city-monthly-avg-retail-price-food/cn-retail-price-36-city-avg-milk-powder-infant-formula-stage-3-400g-pack-import [Accessed September 24, 2024] (22).

## Central Agency for Public Mobilization and Statistics (CAPMAS). Arab Republic of Egypt - Health Survey for the Egyptian Households 2021 (2023). https://censusinfo.capmas.gov.eg/Metadata-en-v4.2/index.php/catalog/665 [Accessed July 11, 2024] (8).

*International Food Policy Research Institute. Fresh Food Price Analysis in Papua New Guinea (2020). <https://www.ifpri.org/project/fresh-food-price-analysis-papua-new-guinea/> [Accessed January 4, 2025] (23).

**The International Food Policy Research Institute. Food price shocks and diets among poor households in Egypt (2022). https://www.ifpri.org/blog/food-price-shocks-and-diets-among-poor-households-egypt/ [Accessed September 24, 2024] (24).

**Supplementary Table S3:** Disability weights for anaemia among children aged 6 months to 24 months

| **Type of anaemia** | **Disability weight** |
| --- | --- |
| **Mild iron-deficiency anaemia** | 0.004 (0.001–0.008) |
| **Moderate iron-deficiency anaemia** | 0.052 (0.034–0.076) |
| **Severe iron-deficiency anaemia** | 0.149 (0.101–0.209) |

**Supplementary Table S4:** Post-hoc analysis (Bonferroni correction)

|  | Bonferroni (dependent variable) | | Mean difference (I-J) | Std. error | Sig. | 95% Confidence interval | |
| --- | --- | --- | --- | --- | --- | --- | --- |
|  |  |  |  |  |  | Lower bound | Upper bound |
| Age (months) | 6 to <12 | 12 to <18 | 0.009 | 0.098 | 1.000 | −0.227 | 0.245 |
|  |  | 18 to <24 | −0.364 | 0.097 | 0.001 | −0.596 | −0.132 |
|  | 12 to <18 | 6 to <12 | −0.009 | 0.098 | 1.000 | −0.245 | 0.227 |
|  |  | 18 to <24 | −0.373 | 0.091 | 0.000 | −0.591 | −0.156 |
|  | 18 to <24 | 6 to <12 | 0.364 | 0.097 | 0.001 | 0.132 | 0.596 |
|  |  | 12 to <18 | 0.373 | 0.091 | 0.000 | 0.156 | 0.591 |
| Wealth index | Lowest quintile  (poorest) | Second quintile (poor) | −0.18 | 0.13 | 1.00 | −0.55 | 0.18 |
|  |  | Third quintile (middle) | −0.27 | 0.12 | 0.28 | −0.62 | 0.08 |
|  |  | Fourth quintile (upper middle) | −0.44 | 0.12 | 0.00 | −0.78 | −0.10 |
|  |  | Highest quintile (wealthiest) | −0.38 | 0.13 | 0.03 | −0.74 | −0.02 |
|  | Second quintile (poor) | 1=Lowest quintile (poorest) | 0.18 | 0.13 | 1.00 | −0.18 | 0.55 |
|  |  | Third quintile (middle) | −0.09 | 0.12 | 1.00 | −0.43 | 0.25 |
|  |  | Fourth quintile (upper middle) | −0.25 | 0.12 | 0.33 | −0.59 | 0.08 |
|  |  | Highest quintile (wealthiest) | −0.20 | 0.13 | 1.00 | −0.55 | 0.16 |
|  | Third quintile  (middle) | 1=Lowest quintile (poorest) | 0.27 | 0.12 | 0.28 | −0.08 | 0.62 |
|  |  | 2=Second quintile (poor) | 0.09 | 0.12 | 1.00 | −0.25 | 0.43 |
|  |  | Fourth quintile (upper middle) | −0.17 | 0.12 | 1.00 | −0.49 | 0.16 |
|  |  | Highest quintile (wealthiest) | −0.11 | 0.12 | 1.00 | −0.45 | 0.24 |
|  | Fourth quintile  (upper middle) | 1=Lowest quintile (poorest) | 0.44 | 0.12 | 0.00 | 0.10 | 0.78 |
|  |  | 2=Second quintile (poor) | 0.25 | 0.12 | 0.33 | −0.08 | 0.59 |
|  |  | 3=Third quintile (middle) | 0.17 | 0.12 | 1.00 | −0.16 | 0.49 |
|  |  | 5=Highest quintile (wealthiest) | 0.06 | 0.12 | 1.00 | −0.28 | 0.40 |
|  | Highest quintile  (wealthiest) | 1=Lowest quintile (poorest) | 0.38 | 0.13 | 0.03 | 0.02 | 0.74 |
|  |  | 2=Second quintile (poor) | 0.20 | 0.13 | 1.00 | −0.16 | 0.55 |
|  |  | 3=Third quintile (middle) | 0.11 | 0.12 | 1.00 | −0.24 | 0.45 |
|  |  | 4=Fourth quintile (upper middle) | −0.06 | 0.12 | 1.00 | −0.40 | 0.28 |
| Child size | Very large | Larger than average | −0.148 | 0.494 | 1.000 | −1.535 | 1.240 |
|  |  | Average | −0.254 | 0.430 | 1.000 | −1.462 | 0.954 |
|  |  | Smaller than average | −0.210 | 0.441 | 1.000 | −1.448 | 1.029 |
|  |  | Very small | −0.127 | 0.448 | 1.000 | −1.384 | 1.131 |
|  | Larger than average | Very large | 0.148 | 0.494 | 1.000 | −1.240 | 1.535 |
|  |  | Average | −0.106 | 0.251 | 1.000 | −0.812 | 0.599 |
|  |  | Smaller than average | −0.062 | 0.269 | 1.000 | −0.818 | 0.694 |
|  |  | Very small | 0.021 | 0.280 | 1.000 | −0.766 | 0.808 |
|  | Average | Very large | 0.254 | 0.430 | 1.000 | −0.954 | 1.462 |
|  |  | Larger than average | 0.106 | 0.251 | 1.000 | −0.599 | 0.812 |
|  |  | Smaller than average | 0.044 | 0.116 | 1.000 | −0.281 | 0.370 |
|  |  | Very small | 0.128 | 0.140 | 1.000 | −0.266 | 0.521 |
|  | Smaller than average | Very large | 0.210 | 0.441 | 1.000 | −1.029 | 1.448 |
|  |  | Larger than average | 0.062 | 0.269 | 1.000 | −0.694 | 0.818 |
|  |  | Average | −0.044 | 0.116 | 1.000 | −0.370 | 0.281 |
|  |  | Very small | 0.083 | 0.170 | 1.000 | −0.395 | 0.561 |
|  | Very small | Very large | 0.127 | 0.448 | 1.000 | −1.131 | 1.384 |
|  |  | Larger than average | −0.021 | 0.280 | 1.000 | −0.808 | 0.766 |
|  |  | Average | −0.128 | 0.140 | 1.000 | −0.521 | 0.266 |
|  |  | Smaller than average | −0.083 | 0.170 | 1.000 | −0.561 | 0.395 |

**Supplementary Table S5:** Change in prevalence due to mild, moderate, and severe anaemia among boys and girls consuming home‑based foods and iron supplements in the initial 2 years of life

| nTimePoint | Gender | nTotal | Mild_% | Mild_n | Moderate_% | Moderate_n | Severe_% | Severe_n |
| --- | --- | --- | --- | --- | --- | --- | --- | --- |
| 1 | Boys | 2869 | 0.262 | 752 | 0.284 | 814 | 0.042 | 121 |
| 2 | Boys | 2869 | 0.265 | 761 | 0.232 | 667 | 0.059 | 169 |
| 3 | Boys | 2869 | 0.257 | 736 | 0.201 | 576 | 0.067 | 193 |
| 4 | Boys | 2869 | 0.247 | 709 | 0.179 | 514 | 0.07 | 202 |
| 5 | Boys | 2869 | 0.235 | 673 | 0.163 | 469 | 0.072 | 207 |
| 6 | Boys | 2869 | 0.222 | 637 | 0.152 | 437 | 0.071 | 205 |
| 7 | Boys | 2869 | 0.212 | 608 | 0.141 | 405 | 0.07 | 202 |
| 8 | Boys | 2869 | 0.203 | 583 | 0.131 | 375 | 0.07 | 200 |
| 9 | Boys | 2869 | 0.195 | 559 | 0.122 | 351 | 0.069 | 197 |
| 10 | Boys | 2869 | 0.187 | 536 | 0.116 | 333 | 0.067 | 192 |
| 11 | Boys | 2869 | 0.178 | 512 | 0.112 | 320 | 0.065 | 187 |
| 12 | Boys | 2869 | 0.17 | 488 | 0.108 | 310 | 0.063 | 182 |
| 13 | Boys | 2869 | 0.164 | 470 | 0.104 | 297 | 0.062 | 177 |
| 14 | Boys | 2869 | 0.158 | 453 | 0.1 | 288 | 0.059 | 170 |
| 15 | Boys | 2869 | 0.154 | 441 | 0.097 | 277 | 0.058 | 165 |
| 16 | Boys | 2869 | 0.149 | 428 | 0.094 | 269 | 0.055 | 159 |
| 17 | Boys | 2869 | 0.145 | 417 | 0.091 | 262 | 0.053 | 153 |
| 18 | Boys | 2869 | 0.143 | 409 | 0.089 | 256 | 0.051 | 147 |
| 19 | Boys | 2869 | 0.14 | 402 | 0.087 | 249 | 0.05 | 143 |
| 20 | Boys | 2869 | 0.137 | 392 | 0.085 | 244 | 0.049 | 141 |
|  |  |  |  |  |  |  |  |  |
| 1 | Girls | 2106 | 0.284 | 599 | 0.274 | 576 | 0.038 | 79 |
| 2 | Girls | 2106 | 0.279 | 587 | 0.227 | 478 | 0.055 | 116 |
| 3 | Girls | 2106 | 0.266 | 561 | 0.194 | 409 | 0.066 | 140 |
| 4 | Girls | 2106 | 0.25 | 527 | 0.177 | 372 | 0.069 | 146 |
| 5 | Girls | 2106 | 0.235 | 495 | 0.16 | 338 | 0.072 | 152 |
| 6 | Girls | 2106 | 0.219 | 462 | 0.151 | 318 | 0.071 | 149 |
| 7 | Girls | 2106 | 0.207 | 436 | 0.141 | 296 | 0.069 | 146 |
| 8 | Girls | 2106 | 0.198 | 417 | 0.132 | 278 | 0.067 | 142 |
| 9 | Girls | 2106 | 0.189 | 399 | 0.123 | 260 | 0.067 | 141 |
| 10 | Girls | 2106 | 0.184 | 388 | 0.116 | 244 | 0.066 | 139 |
| 11 | Girls | 2106 | 0.179 | 378 | 0.109 | 229 | 0.065 | 137 |
| 12 | Girls | 2106 | 0.173 | 365 | 0.104 | 219 | 0.063 | 133 |
| 13 | Girls | 2106 | 0.167 | 351 | 0.1 | 211 | 0.061 | 129 |
| 14 | Girls | 2106 | 0.16 | 337 | 0.098 | 207 | 0.059 | 125 |
| 15 | Girls | 2106 | 0.155 | 327 | 0.094 | 198 | 0.058 | 123 |
| 16 | Girls | 2106 | 0.149 | 314 | 0.092 | 193 | 0.057 | 121 |
| 17 | Girls | 2106 | 0.145 | 306 | 0.088 | 185 | 0.057 | 121 |
| 18 | Girls | 2106 | 0.142 | 298 | 0.085 | 180 | 0.057 | 120 |
| 19 | Girls | 2106 | 0.138 | 290 | 0.084 | 176 | 0.056 | 118 |
| 20 | Girls | 2106 | 0.135 | 284 | 0.083 | 175 | 0.055 | 116 |

**Supplementary Table S6:** Change in prevalence due to mild, moderate, and severe anaemia among boys and girls consuming home‑based foods alone

| nTimePoint | Gender | nTotal | Mild_% | Mild_n | Moderate_% | Moderate_n | Severe_% | Severe_n |
| --- | --- | --- | --- | --- | --- | --- | --- | --- |
| 1 | Boys | 48215 | 0.252 | 12164 | 0.273 | 13177 | 0.044 | 2121 |
| 2 | Boys | 48215 | 0.257 | 12401 | 0.229 | 11029 | 0.063 | 3047 |
| 3 | Boys | 48215 | 0.253 | 12218 | 0.201 | 9692 | 0.074 | 3587 |
| 4 | Boys | 48215 | 0.246 | 11860 | 0.183 | 8813 | 0.081 | 3895 |
| 5 | Boys | 48215 | 0.237 | 11448 | 0.17 | 8206 | 0.084 | 4056 |
| 6 | Boys | 48215 | 0.229 | 11051 | 0.161 | 7758 | 0.086 | 4130 |
| 7 | Boys | 48215 | 0.222 | 10681 | 0.154 | 7419 | 0.086 | 4142 |
| 8 | Boys | 48215 | 0.215 | 10346 | 0.148 | 7138 | 0.085 | 4122 |
| 9 | Boys | 48215 | 0.209 | 10060 | 0.143 | 6903 | 0.085 | 4075 |
| 10 | Boys | 48215 | 0.204 | 9814 | 0.139 | 6698 | 0.083 | 4011 |
| 11 | Boys | 48215 | 0.199 | 9605 | 0.135 | 6510 | 0.082 | 3942 |
| 12 | Boys | 48215 | 0.196 | 9427 | 0.131 | 6339 | 0.08 | 3867 |
| 13 | Boys | 48215 | 0.192 | 9269 | 0.128 | 6194 | 0.078 | 3783 |
| 14 | Boys | 48215 | 0.189 | 9132 | 0.126 | 6057 | 0.077 | 3703 |
| 15 | Boys | 48215 | 0.187 | 9018 | 0.123 | 5924 | 0.075 | 3625 |
| 16 | Boys | 48215 | 0.185 | 8903 | 0.121 | 5810 | 0.074 | 3548 |
| 17 | Boys | 48215 | 0.183 | 8813 | 0.118 | 5701 | 0.072 | 3474 |
| 18 | Boys | 48215 | 0.181 | 8728 | 0.116 | 5598 | 0.071 | 3401 |
| 19 | Boys | 48215 | 0.180 | 8655 | 0.114 | 5505 | 0.069 | 3333 |
| 20 | Boys | 48215 | 0.178 | 8583 | 0.112 | 5419 | 0.068 | 3267 |
|  |  |  |  |  |  |  |  |  |
| 1 | Girls | 46810 | 0.265 | 12384 | 0.257 | 12025 | 0.04 | 1873 |
| 2 | Girls | 46810 | 0.264 | 12354 | 0.218 | 10182 | 0.058 | 2728 |
| 3 | Girls | 46810 | 0.257 | 12020 | 0.193 | 9024 | 0.069 | 3240 |
| 4 | Girls | 46810 | 0.247 | 11580 | 0.176 | 8253 | 0.076 | 3543 |
| 5 | Girls | 46810 | 0.238 | 11126 | 0.165 | 7713 | 0.079 | 3714 |
| 6 | Girls | 46810 | 0.229 | 10699 | 0.156 | 7312 | 0.081 | 3802 |
| 7 | Girls | 46810 | 0.22 | 10315 | 0.15 | 7003 | 0.082 | 3830 |
| 8 | Girls | 46810 | 0.213 | 9982 | 0.144 | 6749 | 0.082 | 3823 |
| 9 | Girls | 46810 | 0.207 | 9705 | 0.14 | 6531 | 0.081 | 3790 |
| 10 | Girls | 46810 | 0.202 | 9464 | 0.136 | 6343 | 0.08 | 3743 |
| 11 | Girls | 46810 | 0.198 | 9256 | 0.132 | 6174 | 0.079 | 3686 |
| 12 | Girls | 46810 | 0.194 | 9076 | 0.129 | 6023 | 0.077 | 3623 |
| 13 | Girls | 46810 | 0.191 | 8927 | 0.126 | 5886 | 0.076 | 3555 |
| 14 | Girls | 46810 | 0.188 | 8797 | 0.123 | 5759 | 0.074 | 3487 |
| 15 | Girls | 46810 | 0.185 | 8682 | 0.121 | 5645 | 0.073 | 3417 |
| 16 | Girls | 46810 | 0.183 | 8586 | 0.118 | 5534 | 0.072 | 3352 |
| 17 | Girls | 46810 | 0.181 | 8492 | 0.116 | 5441 | 0.07 | 3284 |
| 18 | Girls | 46810 | 0.18 | 8415 | 0.114 | 5343 | 0.069 | 3224 |
| 19 | Girls | 46810 | 0.178 | 8344 | 0.112 | 5261 | 0.068 | 3162 |
| 20 | Girls | 46810 | 0.177 | 8282 | 0.111 | 5179 | 0.066 | 3105 |

**Supplementary Table S7:** Health benefits of iron fortification or iron supplementation among infants in early life years (2,13-18)

| **Study Reference** | **Intervention** | **Intervention groups** | **Duration** | **Percent change in anaemia,  % (n/N)** | **Hb change (g/dL), 95% CI** | **Change in iron level (µg/dL), 95% CI** |  |
| --- | --- | --- | --- | --- | --- | --- | --- |
| 1. **On anaemia prevalence, haemoglobin changes, and iron levels** | | | | | | | |
| (14) | Supplements | Iron supplements | 4 months | 57.5 (23/40) | 1.49 (1.13–1.84) | 0.21 (0.08, 0.35) |  |
|  | Supplements | Iron and folic acid supplements | 4 months | 69.1 (29/42) | 1.52 (1.13–1.91) | 0.15 (0.04, 0.26) |  |
|  | Supplements | Multiple micronutrient supplements | 4 months | 72 (26/36) | 1.41 (1.05–1.76) | 0.23 (0.04, 0.42) |  |
|  | Fortification | Fortified water with iron, zinc, and ascorbic acid | 4 months | 52.08 (25/48) | 1.07 (0.77–1.36) | 0.19 (0.06, 0.31) |  |
| (18) | Fortification | Micronutrient-fortified infant cereal | 8 months | 62.4 (63/101) | 1.9±0.20 [mean (g/dL) ± SD] | -- |  |
| (15) | Fortification | IFC | 12 months | 23.4 (15/64) | -- | -- |  |
|  |  | Rice-based iron cereal | 12 months | 45 (36/80) | -- | -- |  |
| 1. **On the growth of children** | | | | | | | |
| **Author ID, year** | **Intervention** | **Intervention groups** | **Duration** | **Weight for age (kg) (mean ± SD)** | **Height for age (cm) (mean ± SD)** | **BMI for age** |  |
| (17) | Fortification | Wheat-based infant cereal | 6 months | 11.8±2.10 | -- | -- |  |
| (16) | Supplements | Ferrous sulphate + zinc + ascorbic acid | 2 years | 0.40±0.98 (difference compared with placebo) | 0.28±0.81 (difference compared with placebo) | -- |  |
| 1. **On cognitive abilities and immunity of children** | | | | | | |  |
| **Author ID, year** |  |  |  | **Productivity loss (USD)** | **Learning disabilities** | **IRR** |  |
| (2) | Fortification | Micronutrient-fortified infant cereal | 9 months | 2005 |  |  |  |
| (15) | Fortification | Iron-fortified cereal | 6 months |  | 107.2±11.6 (Bayley-III for language development in the intervention group) |  |  |
| (13) | Fortification | Iron-fortified formula + cow milk | 18 months |  |  | IRR 0.91 (0.84, 0.98) |  |

BMI: Body metabolic index; CI: Confidence interval; cm: Centimetre; Hb: Haemoglobin; IFC: Iron-fortified cereals; IRR: Infection rate ratio; kg: Kilogram; SD: Standard deviation; USD: United State dollar.


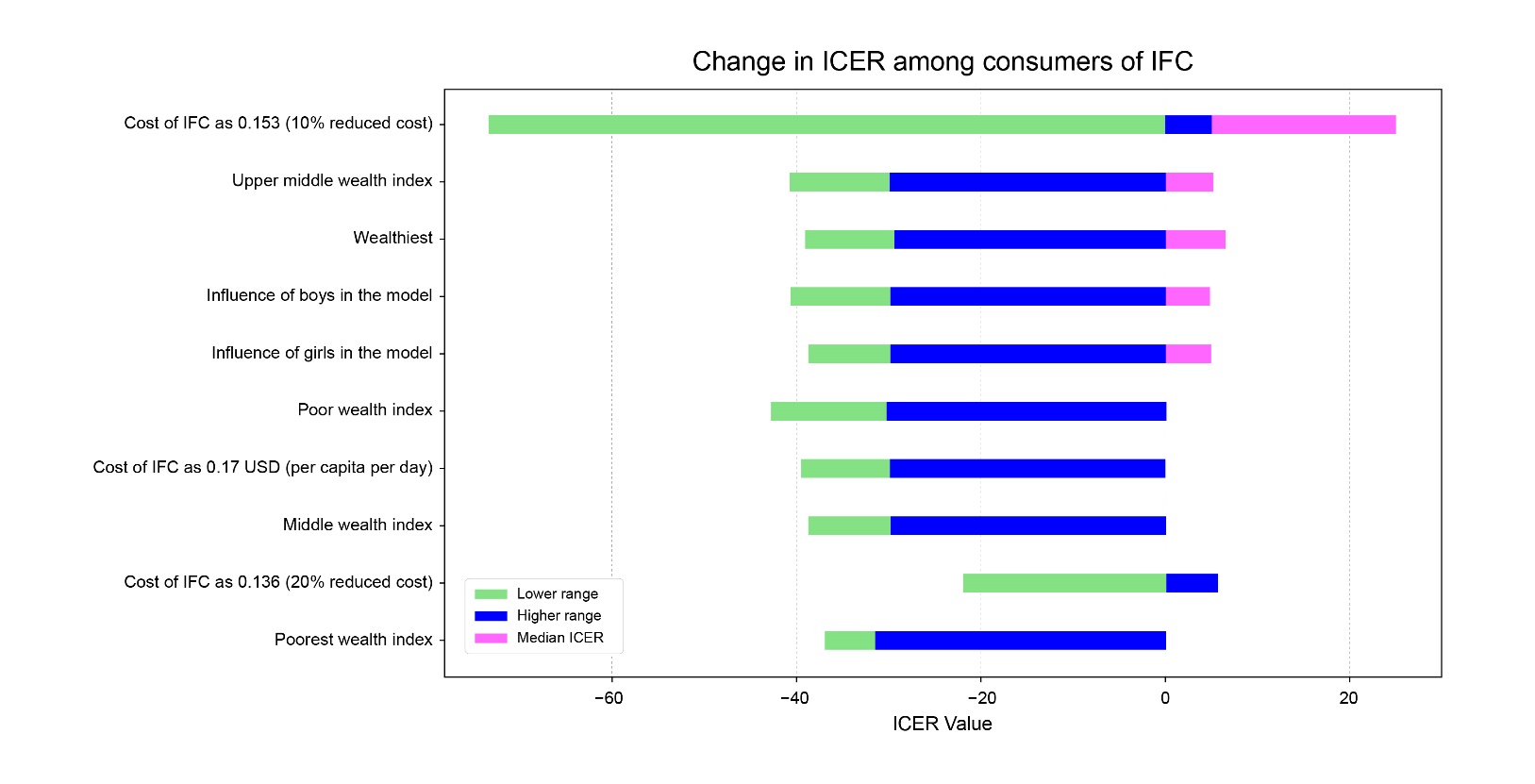


**Figure S1a:** Results from the sensitivity analysis (tornado diagram).


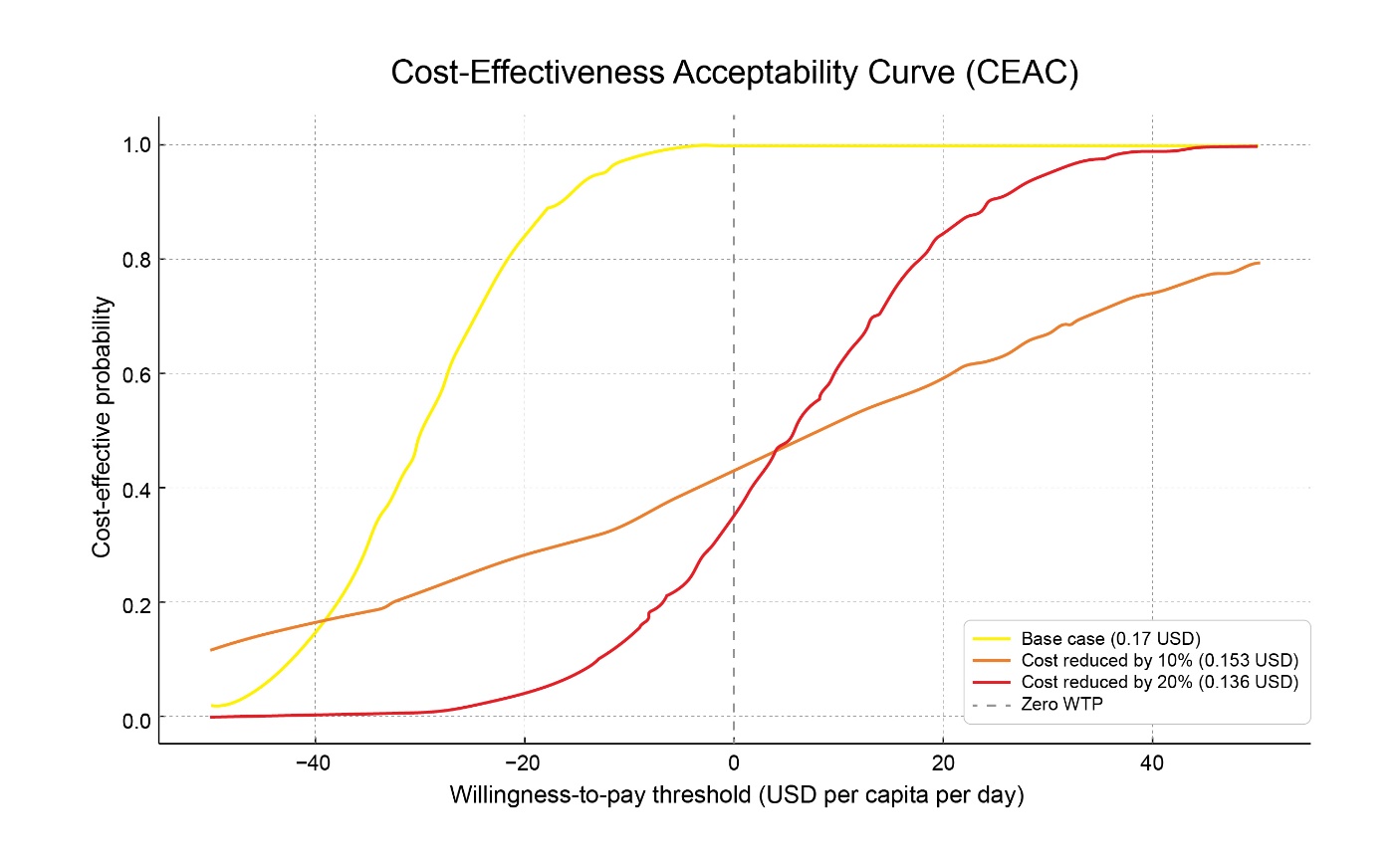


**Figure S1b: Results from the sensitivity analysis CEAC.**

**CEAC: Cost effectiveness acceptability curve**

This CEAC illustrates that IFCs are cost-effective across varying willingness-to-pay (WTP) thresholds. At the base-case (0.17 USD per capita per day), the intervention has an 80% probability of being cost-effective. This probability increases with cost reduction, which reaches around 90%–95% when costs are lowered by 10% (0.153 USD) or 20% (0.136 USD), demonstrating that affordability enhances the intervention’s economic appeal. Under a zero WTP scenario, the probability drops to 40%, emphasising the importance of budget allocation in decision-making. The analysis supports IFC as a viable, cost-effective strategy for addressing iron deficiency in public, particularly when supported by funding mechanisms or cost-saving measures.
